# Integrating succession and community assembly perspectives

**DOI:** 10.12688/f1000research.8973.1

**Published:** 2016-09-12

**Authors:** Cynthia Chang, Janneke HilleRisLambers

**Affiliations:** 1 Division of Biology, School of STEM, University of Washington-Bothell, Bothell, WA, USA; 2 Department of Biology, University of Washington, Seattle, Seattle, WA, USA

**Keywords:** Succession, community assembly, dynamics

## Abstract

Succession and community assembly research overlap in many respects, such as through their focus on how ecological processes like dispersal, environmental filters, and biotic interactions influence community structure. Indeed, many recent advances have been made by successional studies that draw on modern analytical techniques introduced by contemporary community assembly studies. However, community assembly studies generally lack a temporal perspective, both on how the forces structuring communities might change over time and on how historical contingency (e.g. priority effects and legacy effects) and complex transitions (e.g. threshold effects) might alter community trajectories. We believe a full understanding of the complex interacting processes that shape community dynamics across large temporal scales can best be achieved by combining concepts, tools, and study systems into an integrated conceptual framework that draws upon both succession and community assembly theory.

## Introduction and context

**Figure 1.**
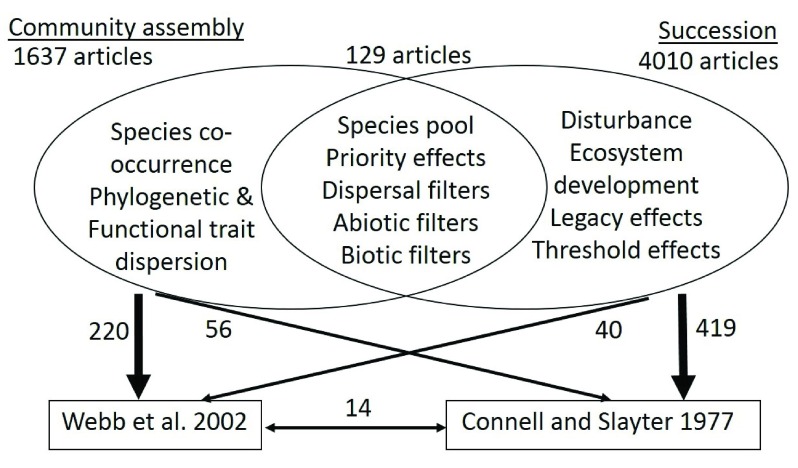
Examining published papers (as searched on Web of Science) that use “community assembly” versus “succession” as key words shows little cross-pollination. Venn diagram depicts typical associated concepts and shows that few articles use both key words. We chose one highly cited “community assembly” paper ^42^ and one highly cited “succession” paper ^2^; both papers are equally cited (~1000 each), with only 14 papers citing both. Of the “community assembly” papers, 220 cited Webb *et al*. ^42^ and 56 cited Connell and Slayter ^2^. Of the “succession” papers, 419 cited Connell and Slayter ^2 ^and 40 cited Webb *et al*. ^42^.

**Figure 2.**
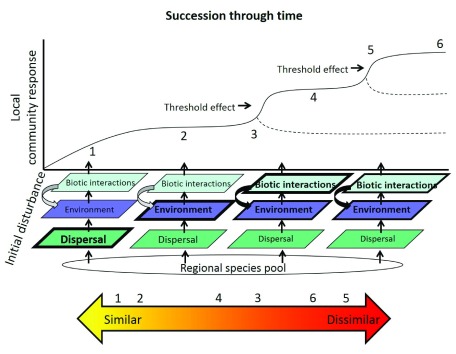
Integrated conceptual framework of community assembly processes over the course of succession after a disturbance. The regional species pool undergoes a filtering effect over the course of succession, where different filter effects are hypothesized to be more important at different time points (different size and bold of text indicates hypothesized relative strength). Threshold effects are driven by complex interactions and feedbacks that determine local community response (changes diversity, composition, and/or functional trait values) and shifts the community into an alternative state. Dotted lines represent alternative community states. Numbers represent changes in community composition or functional trait similarity across a gradient (yellow to orange arrow). Early in succession (1 and 2), communities start off more similar owing to dispersal and environmental filtering effects. At a threshold (3 and 5), the community becomes dissimilar in a short time span. As succession progresses, biotic interactions and environmental feedbacks make communities increasingly dissimilar compared to earlier in succession (4 and 6).

An integrated conceptual framework that incorporates both traditional theories of succession and contemporary community assembly models will provide a holistic understanding of how communities change through time. Succession studies, a foundational ecological topic with a long history and large body of published literature ^1– 4^, provide the opportunity to understand community assembly from a known starting point and as a dynamic process integrated over long periods of time and across large spatial scales ^5^. Likewise, more recently developed community assembly frameworks and associated analytical tools have the potential to bring new insights into our understanding of ecological succession ^5^. Currently, there is insufficient cross-pollination between these related subfields in ecology ( Figure 1). The purpose of this review is to A) compare and contrast classic successional theory with more recent community assembly theory, B) provide an overview of recent advances in succession research that harnesses community assembly conceptual frameworks and tools, and C) provide suggestions on how studies of community assembly might better make use of traditional successional concepts and datasets to develop an integrated framework of succession and community assembly dynamics ( Figure 2).

## Succession and community assembly theory

Succession and community assembly research have clear links in the field of ecology ( Figure 1) ^2, 6, 7^. Both draw from fundamental ecological fields, including biogeography ^8, 9^, phylogenetics and evolution ^8, 10, 11^, trait-based ecology ^10, 12^, and coexistence theory ^13^, and therefore have many overlapping concepts ( Figure 1). For example, community assembly and successional studies share a focus on the importance of dispersal, environment, biotic filters, and stochastic events. For this reason, modern analytical and conceptual frameworks pushed forward by community assembly research have recently been applied to long-term successional datasets ^5^, and these new tools have clearly pushed forward our understanding of successional processes after a disturbance.

However, there are differences in the perspective the two fields take on community dynamics and thus which processes are emphasized in studies. Broadly speaking, succession research is rooted in studies that describe the development and trajectory of communities and (often) ecosystems over time after a known disturbance (defined as removal of biomass from abiotic or biotic forces) ^1, 14^. By contrast, community assembly studies examine the rules and mechanisms that dictate local diversity patterns formed from a regional species pool ^2, 6, 7, 15^ regardless of disturbance history, and generally do not consider how community level patterns influence ecosystem processes and vice versa. Thus, the most obvious difference between succession and community assembly research lies in their temporal perspective on community development, especially as it relates to disturbance. Most successional studies begin immediately after disturbance, while community assembly studies, by contrast, use diversity patterns from a single (or short) timespan to infer past processes and mechanisms ^16– 19^. Succession studies place the processes that influence community structure (i.e. assembly mechanisms, also called filters) in an explicitly temporal context, where the relative importance of these different filters may vary over time ( Figure 2). The temporal perspective that successional studies take also emphasizes the importance of priority effects ^20, 21^, legacy effects ^22, 23^, and other stochastic processes such as rare events (e.g. climate extremes and disease/herbivore/pest outbreaks) that have the potential to alter community trajectories ^24– 27^. Succession studies more often acknowledge threshold dynamics ^28^, which occur when abiotic perturbations allow complex, positive feedbacks to occur, causing the system to rapidly change and become a new, alternative state ^28^. In summary, long-term succession research differs from community assembly research in its focus on determining how the processes that shape community trajectories vary in importance over longer periods of time ( Figure 2), how these processes may interact, and the role of stochastic mechanisms such as priority effects, legacy effects, and complex positive feedbacks.

## Major recent advances in succession research using a community assembly framework

Classic succession studies involved studying primary and secondary succession after a disturbance using experiments ^29^, conceptual ^2^ and simulation models ^6^, and observational studies ^2, 30– 32^ through time to understand how communities develop and change through time ^2, 33^. Many of these studies examined rates of succession across disturbance gradients ^34, 35^ and either focused on key successional species ^29, 36^ or compared changes in community types over time ^37– 39^. Recently, long-term succession studies have begun to test hypotheses regarding which stochastic and deterministic processes are more important in early versus late succession by applying recently developed analytical tools developed in the community assembly literature ^5^. For example, it is hypothesized that dispersal generally plays the strongest role immediately following a disturbance and dictates the types of organisms that are able to colonize and spread initially ^2, 15^ ( Figure 2). Similarly, abiotic factors (i.e. environmental filters) are thought to play a strong role early in succession ^15, 40^, with biotic interactions such as competition and facilitation becoming increasingly more important as succession progresses ^41^ ( Figure 2). Null model phylogenetic and functional trait dispersion analyses are quantitative approaches introduced by community assembly studies that have allowed for tests of these hypotheses by assessing whether communities comprise species that are phylogenetically or functionally more different or similar to each other ^10, 42, 43^ as compared to a simulated null model of community assembly. Over-dispersed patterns are assumed to be caused by competition and niche differentiation ^15^. Under-dispersed (clustered) patterns can be explained by either environmental filtering effects or competitive exclusion, though distinguishing between these mechanisms requires additional knowledge about the biology of the system ^44^.

Studies applying these approaches have generally found support for these hypotheses, with some important nuances. For example, older successional communities in tropical forests and grassland chronosequences tend to exhibit phylogenetic over-dispersion – implying biotic interactions ^45– 48^. Similarly, communities generally became more phylogenetically over-dispersed over the course of succession in the volcanic primary successional habitat, although this pattern did not hold across the disturbance gradient ^49^. On the other hand, Li *et al*. used 44 years of old-field primary successional data and found that it is species colonization early in succession and not competitive exclusion that ultimately drives phylogenetic and functional trait over-dispersion and plant community diversity patterns ^50, 51^. All of these studies applied null model analyses to long-term succession data and therefore provided insight into the relative importance of potential filters over time.

Trait-based analyses commonly used in community assembly studies have also provided a more detailed, mechanistic understanding of how niche differences, functional traits, and general ecological strategies interact over the course of succession ^40, 52^. Lebrija-Trejos *et al*. used community trait changes in secondary successional forests to illustrate the directional changes related to environmental filtering effects early in succession ^53^. After examining functional leaf traits related to photosynthetic capacity, as well as water and heat stress tolerance, Lohbeck *et al*. found that in tropical successional forests, species abundant early in succession had more similar traits, while dominant species were more functionally dissimilar later in succession ^54^. Similarly, Lasky *et al*. used tree demography data in a secondary successional tropical forest and found that, as succession progressed, tree communities had higher trait diversity in leaf traits associated with competitive ability (specific leaf area and leaf dry matter content ^55^) and lower trait diversity in traits associated with survival (wood specific gravity) ^56^. In volcanic primary successional plant communities, dispersal traits became more dissimilar over the course of succession and were more dissimilar in disturbance sites closer to intact seed source ^49^. However, competitive versus stress tolerance, nutrient acquisition, and herbivore resistance traits showed no consistent patterns over time or across the disturbance gradient, suggesting that species interactions can be complex and not necessarily generalizable across larger spatial scales ^49^. Finally, studying different traits in a community assembly context has also provided a more holistic, integrated understanding of how different assembly filters could operate on different aspects of species functional variation. For example, in long-term ecosystem development in a Western Australian sand dune chronosequence, plant leaf traits showed convergence towards high nutrient use efficiency in a low-nutrient environment ^57^. However, the same plant communities in low-nutrient environments showed high functional diversity (community dispersion) in belowground nutrient acquisition traits, possibly suggesting biotic filters promoting niche differentiation in belowground nutrient acquisition strategies ^58^.

## Future directions: building an integrated framework to understand succession and community assembly dynamics 

Community assembly studies in ecology have dramatically increased over recent years, largely because new analytical tools allow us to tackle broad questions about community dynamics and coexistence. Surprisingly, despite the clear conceptual links to succession, these recent community assembly studies have not always taken full advantage of the classic successional concepts and studies (but see examples ^5, 50, 54, 56, 57, 59^). Although a partial explanation for this is that long-term successional datasets are often difficult to collect, such data could provide the unique ability to test hypotheses about community assembly processes over time with a known disturbance history. Below, we provide examples for how succession and community assembly research can be integrated to provide a holistic understanding of mechanisms that shape changes in the patterns of community diversity over time across large temporal and spatial scales ( Figure 2).

Long-term successional studies across environmental and/or disturbance gradients provide unique opportunities to assess how these gradients influence a myriad of processes of interest to community assembly. Additionally, they provide context for linking plant and biogeochemical development to community assembly mechanisms ^4^. For example, Laliberté *et al*. found that environmental filtering from the regional species pool was linked to long-term soil development and not local biotic and abiotic filters such as resource competition-dictated plant diversity patterns along a sand dune chronosequence in Western Australia ^59^. Similarly, Mason *et al*. were able to relate community trait dispersion pattern changes over the course of succession to soil ecosystem development in a New Zealand chronosequence in cool temperate rainforest ^60^. Here, the authors found support for the stress-gradient hypothesis ^61^, which suggests that in a high-resource environment, species are more competitive and thus should be more functionally differentiated, as opposed to in a low-resource environment where species are more stress tolerant and thus converge on functional traits that allow them to retain limiting resources ^60^. These studies are examples of how incorporating broad spatiotemporal scales across environmental or disturbance gradients are key to providing insight into how macroevolutionary processes (biogeographical determination of regional species pool) and microevolutionary processes are determined by the local selective abiotic and biotic environment ^4^. Observational successional studies paired with experimental tests could provide a powerful framework to disentangle various abiotic versus biotic filtering effects on long-term species coexistence (a key focus in community assembly studies) ^62^. In addition, future succession studies that determine survival rates of species with and without competitors/facilitators across an environmental/disturbance gradient or resource manipulation could provide key insight into the relative importance of community assembly mechanisms over time.

Long-term succession studies are also ideal for assessing the importance of stochastic events such as priority effects during the community assembly process. Certainly, experimental approaches have already been used to demonstrate that priority effects (i.e. species colonization order) can dictate subsequent community trajectory ^20, 21, 26, 63^. However, successional studies can provide complementary examples of when and how priority effects drive community assembly via positive or negative niche modification (when the first species to arrive modifies the niche, impacting subsequent species assembly) or niche pre-emption (when the first species to arrive occupies a specific niche) ^20^. For example, in old-field succession, legacy effects and priority effects via plant-soil feedbacks exhibit niche modification (sensu ^20^) and dictate early succession processes ^64^. In volcanic primary succession, the arrival of a nitrogen-fixing plant facilitated the growth of other plant species, increasing the rate of succession by increasing soil nitrogen conditions in areas where it successfully colonized and spread ^65, 66^. In marine and aquatic ecosystems, post-disturbance species arrival order can alter community trajectories via niche pre-emption where earlier species can outcompete or dominate a resource before later arrivals ^67^, as well as via niche modification, which sometimes creates alternative stable states within the ecosystem ^68, 69^. Although such priority effects have been demonstrated experimentally in community assembly studies, the long-term effects of alternative community trajectories on community and ecosystem development rates and the mechanisms that drive these processes can be more fully explored in successional studies.

Knowledge of past disturbance history, a clear focus of succession studies, can also provide necessary context for understanding legacy effects, where residual habitat/environmental conditions drive community assembly processes ^70^. For example, secondary succession in forest communities is often defined by residual, intact, partially living trees or fallen debris that provide spatial diversity across the landscape and dictate subsequent local community trajectories ^22, 71^. On Mount St. Helens, secondary succession forests were impacted by residual legacy effects in the blowdown zone following a major volcanic eruption, where scorched, fallen, dead trees created a heterogeneous habitat for the recolonizing plant community by providing nutrient additions and physical structural changes that led to the accumulation of snow in the winter and additional shade in the summer ^71, 72^. Decades after the original fallen trees had mostly decomposed, their residual effects could be seen in the subsequent reassembled community ^71, 72^. There are also many examples of refugia (legacy soil and surviving insect/animal populations) playing a lasting role in succession on Mount St. Helens ^73^ and other primary successional habitats ^74^. Together, these studies highlight the importance of disturbance history context when inferring community assembly mechanisms from observed patterns.

Using only present day diversity patterns without disturbance history knowledge or long-term succession data to understand community assembly processes may be misleading about the importance of abiotic filters driven by stochastic processes. Similar to stochastic, abiotic factors that lead to spatial heterogeneity (e.g. refugia areas), there can also be temporal heterogeneity impacting community trajectories owing to events such as climate extremes ^24, 27^, nutrient pulse events ^25^, or abnormally high herbivore/disease load. These (potentially) rare events require long-term succession data to evaluate the strength and longevity of impact on community assembly. Kreyling *et al*. found that extreme climate events caused lasting stochastic changes in the plant community succession in experimental grassland communities ^24^. In microbial community assembly, Zhou *et al*. found that stochastic processes controlled community succession in a dynamic system following a high nutrient amendment ^25^, whereas in aquatic pond ecosystems, Chase found that stochastic processes were stronger in non-drought assembled communities compared to drought ^27^. In an era of rapid global change with an increasing number of extreme and/or disturbance events, the fact that many species and species interactions are characterized by lag-times ^75^ highlights the high value of long-term succession studies in understanding community structure and assembly.

Finally, a hallmark of succession is the idea that non-equilibrium states caused by disturbances consistently lead to community reassembly in a scale-dependent manner ^1, 76– 78^. These threshold or regime shifts are characterized by an ecosystem that undergoes an abrupt change caused by abiotic and/or biotic drivers ^28, 76^. Classic examples of threshold dynamics in succession are found in the transition from grasslands to shrub-/woodland-dominated ecosystems driven by natural disturbance mechanisms like fire and grazing ^79^, as well as in forest ecosystems where dominant species composition changes over time ^80^. Looking at threshold effects allows us to disentangle complex feedback mechanisms that lead to these alternative states and provides a more nuanced framework to understanding community assembly. Threshold transitions provide mechanistic insight into feedbacks between abiotic and biotic factors ( Figure 2, black circular arrows) and, if done across an environmental or disturbance gradient, has the potential to provide insight into alternative stable states depending on subsequent species assemblages. Threshold models and succession theory have been applied to a restoration context ^28^ and highlight the necessity for integrating the mechanistic understanding of community assembly and successional dynamics that have occurred over long periods of time after a disturbance event.

## Conclusions

Recent advances and growing attention in community assembly research have allowed ecology to make great strides with unique insights into the processes that dictate community species diversity patterns. However, it is important not to overlook the foundational conceptual frameworks built on classic successional studies. The growing wealth of long-term successional datasets coupled with the advances in robust analytical tools will allow succession and community assembly research to address many challenges facing ecosystems around the world. Specifically, classic successional research has emphasized disturbance, alternative community trajectories, and temporal dynamics, all of which are critical to understanding how communities assemble and disassemble in response to global change. Moreover, succession datasets are particularly poised to inform restoration, land management, and conservation goals. Combining observational and experimental approaches in an integrated framework will allow us to understand how communities and ecosystems respond to natural and anthropogenic disturbance and global changes in our future environment.
